# Can nudging increase the uptake of recommended health services?

**DOI:** 10.1016/j.pecinn.2026.100508

**Published:** 2026-09-26

**Authors:** Ole Kristian Aars, Tor Iversen

**Affiliations:** a The Norwegian Medical Products Agency, Oslo, Norway; b Department of Health Management and Health Economics, University of Oslo, Oslo, Norway

**Keywords:** Information, Nudging, Health behavior, Type 2 diabetes, Field experiment

## Abstract

**Objective:**

Type 2 diabetes (T2DM) represents a significant threat to global public health. When supported by their general practitioner (GP), patients who self-manage effectively can prevent or delay serious complications. This study examines whether an innovative information initiative can nudge members of the Norwegian Diabetes Association (NDA) with T2DM to schedule a comprehensive annual checkup with their GP.

**Methods:**

We randomized 12,484 NDA members with T2DM into an intervention group with two subgroups and a control group with two subgroups. The intervention group received a message describing the health benefits of a comprehensive checkup, a link to an explanatory video, and a prompt to contact their GP to book an appointment. One intervention subgroup received an email followed by an SMS reminder 1 week later (Group 1); the other intervention subgroup received both messages by SMS (Group 2). The control group received no information. All groups were sent a questionnaire 4 weeks after the initial message.

**Results:**

The overall effect of the intervention was 3.5 percentage points (*p* = 0.020). Participants in intervention Group 1 initiated a comprehensive annual checkup 4.2 percentage points (*p* = 0.024) more often than the control group. For Group 2, we did not find a statistically significant effect of the intervention.

**Conclusion:**

This intervention nudging patients with T2DM to initiate a comprehensive annual checkup had a significant effect, particularly for the group that received initial information by email and a reminder by SMS.

**Innovation:**

This study adds to the emerging literature on low-cost digital interventions by demonstrating that brief, scalable digital information can increase uptake of guideline-recommended preventive care among patients with T2DM. The findings suggest that such interventions may offer a cost-effective approach to increasing preventive-care uptake, which could in turn help reduce diabetes-related complications at the population level.

## Highlights

• Patients with type 2 diabetes (T2DM) should visit their general practitioners for an annual check-up
• We design and test an intervention to encourage this preventive visit
• We randomized members of Norwegian Diabetes Association to intervention and control
• We find that participants in the intervention group were more likely to attend preventive visits.
• The intervention helps prevent diabetes complications in a cost-effective way.

## 1 Introduction

Diabetes and its complications represent a major threat to global public health in the 21st century. According to the International Diabetes Federation [1], an estimated 589 million adults aged 20–79 years are currently living with type 1 or type 2 diabetes worldwide, of whom approximately 43% remain undiagnosed. In 2024 alone, diabetes was responsible for 3.4 million deaths, and the global burden is projected to rise sharply, with the total number of cases expected to reach 853 million by 2050. Type 2 diabetes mellitus (T2DM) is by far the most prevalent form of the disease, accounting for approximately 90% of all cases. In Norway, T2DM represents one of the largest chronic disease burdens, affecting more than 250,000 people—nearly 5% of the population.

T2DM is a progressive disorder characterized by impaired insulin secretion and action, leading to dysregulated glucose metabolism and related metabolic disturbances [2]. When left untreated, it produces hyperglycemic states and increases the risk of both macrovascular complications — such as myocardial infarction and stroke — and microvascular complications, including retinopathy, nephropathy, and neuropathy. Blood pressure and glycemic monitoring, lipid management (statin therapy) and smoking cessation have all been shown to reduce the risk of macrovascular complications, while glycemic monitoring and blood pressure reduction are particularly important in mitigating microvascular complications.

The cornerstone of T2DM management is a personalized self-management program, typically encompassing healthy nutrition, physical activity, blood glucose and blood pressure monitoring, medication adherence, and other risk-reduction strategies. Alongside effective self-management, general practitioners (GPs) play a pivotal role in screening for disease-related complications and developing individualized preventive care plans.

Because disease management is a shared responsibility between the GP and the patient, it is important to understand why many patients fail to attend and how participation rates can be improved. Patient education is critical for supporting self-management and enabling the early detection of complications. In the present study, we evaluate the effects of an intervention targeting people with T2DM in Norway. The study is inspired by previous work on ways to improve self-management, including in-person education models [3] and web-based educational interventions [4]. Previous studies show that reminder emails and SMS messages can lead to behavioral changes. Although the magnitude of the effect varies, Hasvold and Wootton [5] find that SMS reminders can lead to a 30% increase in appointment attendance, and Samoocha et al. [4] report statistically significant results for emails, albeit with a slightly lower effect. Recent studies have shown that reminders are effective in childhood vaccination [6] and that SMS reminders improve health screening uptake [7]. Other studies have shown that SMS reminders support self-management, including improved adherence to oral medication for T2DM [8], and a reduced occurrence of STDs [9] and HIV [10].

In the literature, interventions as described above are often referred to as empowerment or nudges. Although these concepts are frequently used interchangeably, decisive factors typically relate to whether they target people's actions directly or indirectly. Empowerment is broadly defined as “helping patients discover and develop the inherent capacity to be responsible for one's own life” ([11], p. 38). Empowerment is a *process* or *outcome*, not necessarily a single intervention [11]. This definition differs from the concept of nudging, which involves interventions targeting an individual's choice architecture [12]. Hertwig and Grüne-Yanoff [13] argue that nudging can have an educational component, which they regard as “boosting.” The boundaries between the concepts are therefore not strict. We understand nudging as one of the many instruments that can help people make behavioral changes, whereas empowerment is more comprehensive and often involves interactions between patients and health personnel. For the remainder of the article, we refer only to “nudging” since it better describes our simple intervention.

In Norway, the National Health Service provides healthcare to more than 5 million residents. Norway implemented its regular GP scheme in 2001. Each resident may register with a GP, who provides primary care and acts as a gatekeeper for specialized care. Close to 100% of the population have a regular GP. Approximately 80% of the 5000 Norwegian GPs are private practitioners under contract with a municipality [14]; the remainder are salaried municipal employees.

In 2006, the Norwegian Quality Improvement of Primary Health Care Laboratories (NOKLUS) initiated a program of comprehensive annual checkups by the GP for patients with T2DM together with a national quality register called the Norwegian Diabetes Register for Adults. For data submission, physicians must install computer software (the NOKLUS form) linked to patients' electronic records. The software also assists physicians in organizing comprehensive annual checkups so that all the required components are included. These components comprise management of blood pressure, total cholesterol, low-density lipoprotein, albuminuria, as well as foot and eye examinations. A dedicated fee was introduced to incentivize GPs to conduct the comprehensive annual checkups for patients with T2DM. Despite the recommendation of NOKLUS by the Directorate of Health [15] and a fee for the comprehensive annual checkups and data submission, overall checkup rates among people with T2DM remain low. In 2022, approximately one-third of Norwegian GPs conducted comprehensive annual checkups for their patients with T2DM ([16], p. 26).

A low number of patients with T2DM on a GP's list, combined with a high workload, has been identified as a factor contributing to poorer performance in T2DM follow-up [17]. Bakke et al. [18] conclude that the overall quality of screening procedures is suboptimal and advocate for the mandatory use of a structured diabetes monitoring form. However, less stringent measures may also prove effective, as demonstrated by Iversen and Ma [19].

The aim of the present study is to examine the effect of a demand-side intervention: specifically, the extent to which patients, after receiving a nudge, initiate a comprehensive annual checkup with their GP. The experimental group received information about the benefits of attending a comprehensive annual checkup and was encouraged to contact their GP for one. One subgroup received the information by email, while the other received an SMS (text message). The control group received no information. We hypothesize that participants in the intervention groups increase their attendance relative to the control group as a result of the messages. We make no predictions regarding the relative effects across the intervention subgroups.

The article proceeds as follows. Section 2 presents the methods, Section 3 presents results, and Section 4 discusses the research, summarizes its innovative content, and concludes.

## 2 Methods

This study was conducted in September 2021, in collaboration with the Norwegian Diabetes Association (NDA). The study was assessed by the Regional Committee for Medical and Health Research Ethics, South-East Norway (REK sør-øst A, reference 218,637) on 4 January 2021, prior to commencement. REK decided that the project falls outside the scope of the Health Research Act, as it does not collect individual health data; no formal ethical approval was therefore required. No changes were made to the trial design, eligibility criteria, interventions, or outcomes after the trial commenced.

Based on the available contact information, we identified two strata and randomized members within each stratum into intervention and control arms; in a parallel-group superiority design. The intervention arm in the first group (Group 1) received an email stating the benefits of a comprehensive annual checkup and urging them to consult their GP regarding scheduling, followed by an SMS reminder 1 week later. The intervention arm in the second group (Group 2) received an abbreviated version of the email by SMS, followed by an SMS reminder 1 week later. For both groups, all messages linked to a webpage hosted by the NDA containing an educational video about the annual checkups. This design was motivated by previous studies showing that SMS messages are more effective when coupled with video messages [20]. Two weeks after the reminder messages, online questionnaires were sent to the intervention and control arms of both groups.^1^ The messages introducing the questionnaires followed the same specifications as the interventions for each subgroup, with reminders sent 1 week later.

Some individuals who received the information had likely undergone a comprehensive annual checkup previously. We hypothesized that previous attendance would increase the likelihood of attendance this year because these patients and their GPs were already aware of the advantages of a comprehensive annual checkup and because the patients' GPs were likely to have installed the software that initiates the NOKLUS form.

The NDA is one of the largest patient organizations in Norway, with more than 32,000 members. Approximately 14,000 members disclosed that they have T2DM, and 12,484 of them had provided telephone details to the NDA. Of these, 7813 also provided email addresses. To account for possible bias and ensure balanced samples, we^2^ randomized the control and intervention groups using the contact information available to us. Table 1 shows the randomization process.

**Table 1:** Randomization (based on the availability of contact information).

	Intervention	Control	Sum
Group 1: Email and SMS	3906	3907	7813
Group 2: Only SMS	2335	2336	4671
Sum	6241	6243	12,484

We conducted a power analysis using the Wilcoxon signed-rank test to prepare for the experiment. The analysis indicated that we would likely require a 3.3% intervention effect to generate a statistically significant result at the aggregate level. Given that similar studies have shown modest effects [21], we decided to send a reminder both to increase the chances of a significant result and to mimic real-world settings, in which reminders are commonly used [22].

The intervention consisted of an informational email or SMS (depending on whether individuals were in Groups 1 or 2), followed by an SMS reminder a week later. For Group 1, the first email carried the heading “Have you gone for your annual diabetes checkup?” The text stated that: (i) annual checkups are part of the medical guidelines for people with diabetes, (ii) there are health benefits to the early detection of symptoms, (iii) many GPs do not actively invite their patients, and (iv) encouraged patients to consult their GP if they had not received an invitation. The email also contained a link to a closed NDA webpage; that was inaccessible without the link. The page described the benefits of a comprehensive annual checkup and contained a video in which an NDA employee explained these benefits in greater detail. A short note at the bottom of the page requested that people not disclose the information. The purpose was to avoid contamination of the control group, but it also reflected a stated wish by the NDA, relating to transparency toward its membership. Group 2 received the abbreviated version by SMS.

Online questionnaires were sent to the intervention and control groups 4 weeks after the first intervention. To improve the response rate, respondents could enter a lottery for the chance of winning a gift card worth of NOK 1000 (approximately USD 115 in Septmber 2021). Identical questionnaires were developed so that respondents could be categorized according to i) whether they were in Group 1 (email and SMS) or Group 2 (SMS only) and ii) whether they belonged to the control or intervention group. Using the NDA's email software, we were also able to identify whether an individual had opened the email, and whether they had clicked on the link. We were unable to determine the same for the SMS since link-tracking is not available for SMS.

The survey began by asking whether the respondent had visited or made an appointment with the GP for a checkup during the previous 12 months. Because the intervention took place 4 weeks before the survey was distributed, some visits occurred before the intervention. Since the intervention and control groups were randomized, visits before the intervention are randomized between the groups by construction and pose no threat to the validity of the study.

We obtained four separate but identical datasets for the questionnaire responses. A dummy variable was added to each dataset according to the categorization above before the datasets were merged. This allowed us to identify the responses according to the type of intervention to which respondents had been exposed. A schematic overview of the design is presented in Appendix A Fig. A.1. The response rates were 31% in Group 1 and 22% in Group 2. Response rates were equal in intervention and control arms.

The effect of the intervention is measured by a completed checkup or by arranging for a checkup. We define a binary variable “*Checkup*,” equal to 1, for respondents stating that they (i) had undergone a checkup, (ii) had booked an appointment for a checkup, or (iii) had seen their GP and undergone tests that are normally included in a comprehensive annual checkup. Following advice from the NDA, the third category was included to reflect that some patients may have been unaware that they had undergone a comprehensive annual checkup (see Appendix B for details). Given the nature of the intervention, no physical, psychological, or other harms were anticipated or systematically assessed.

In the pooled sample, we control for the imbalance between Groups 1 and 2 by introducing covariates. We estimate a standard probit model where the likelihood of a binary outcome is contingent on a set of covariates, Z**,** $\mathrm{Prob}\left(Y=1|Z\right)$**.** In the probit model in Eq. (1), there are *j = 4* groups. Group 1.1 is the intervention group that receives an email and an SMS; Group 1.2 is its control group; Group 2.1 is the intervention group that received an SMS; and Group 2.2 is its control group. The index *ij* denotes individual *i* in group *j*. The binary variable *Checkup*_*ij*_ equals 1 if individual *i* in group *j* has a checkup, and 0 otherwise. ${\mathrm{Int}}_{\mathrm{ij}}$ is a binary variable equal to 1 if individual *i* belongs to Groups 1.1 or 2.1, and 0 otherwise. *Previous_checkup*_*ij*_ is equal to 1 if individual *i* in group *j* had checkup previously and zero otherwise. *Male*_*ij*_ is equal to 1 if individual *i* in group *j* is male and 0 otherwise. *Age*_*ij*_ is the age interval of individual *i* in group *j*, and *Diabetes_years*_*ij*_ is the number of years since individual *i* in group *j* was diagnosed with T2DM.

(1) $$\text{Prob}\left({\text{Checkup}}_{\mathrm{ij}}=1|Z\right)=\Phi \left(b_{0}+b_{1}{\mathrm{Int}}_{\mathrm{ij}}+b_{2}\text{Previou}{s_{\text{checkup}}}_{\mathrm{ij}}+b_{3}{\text{Male}}_{\mathrm{ij}}+b_{4}{\mathrm{Age}}_{\mathrm{ij}}+b_{5}\text{Diabetes}\_{\text{years}}_{\mathrm{ij}}\right)$$

$$\left(i=1,\ldots ,n_{j}\right),\left(j=1.1,1.22.12.2\right),$$

where Φ denotes the cumulative distribution function of a standard normal distribution. The parameters *b*_*0*_*,….b*_*5*_ are estimated using maximum likelihood.

Finally, for each of the two subgroups, we test whether the proportion of checkups differs between the intervention and control groups using a chi-square test of independence. All statistical analyses were performed in Stata version 16.

## 3 Results

Descriptive statistics of core attributes of the two subsamples (Groups 1 and 2) show that the observations are not well balanced across subsamples. People in Group 2 were generally older, had been diagnosed with T2DM for longer, and had undergone checkups more often. Details are shown in Table A.1.

Table 2 presents the descriptive statistics for the control and intervention groups for the full sample and the two subsamples. In the full sample, 73% of respondents had previously undergone diabetes-specific annual checkups. This proportion is substantially higher than the national figure referred to in the Introduction, probably because NDA members are more motivated than the average person with T2DM. The proportion is approximately the same in the intervention and control groups. Most respondents were aged 60 years or older and had T2DM for more than 10 years. Within each subsample, the intervention and control groups appear well balanced, as confirmed by an asymptotically normally distributed test statistic and Kolmogorov–Smirnov test. We could not reject the hypothesis that the proportions are equal in the intervention and control groups.

**Table 2:** Descriptives and balance for each subsample.

	Full sample			Group 1: Email and SMS			Group 2: Only SMS		
	Intervention	Control	Diff	Intervention	Control	Diff	Intervention	Control	Diff
Previous checkup	73.5	72.9	0.6	72.1	70.8	1.3	76.8	78.1	−1.3
Male	50	48.8	1.2	50.8	50.3	0.5	47.9	45.2	2.7
Age									
20–39 years	1.4	1	0.4	1.9	1.2	0.7	0.4	0.6	−0.2
40–59 years	19.3	20.5	−1.2	22.7	23.1	−0.4	10.7	13.8	−3.1
60+ years	79.2	78.4	0.8	75.4	75.7	−0.3	88.8	85.5	3.3
Years with T2DM									
<1 year	3.5	3.3	0.2	4.8	4.4	0.4	0.3	0.6	−0.3
1–2 years	8.6	6.8	1.8	10.8	8.4	2.4	3.3	2.8	0.5
3–10 years	22.5	25.5	−3	26.3	28.5	−2.2	13.5	18	−4.5
11+ years	65.3	64.4	0.9	58	58.7	−0.7	82.8	78.5	4.3

Note. Equal distributions across the two groups (Groups 1 vs. Group 2) was rejected using asymptotically normally distributed test statistics and a two-sample Kolmogorov–Smirnov test.

The descriptive statistics and associated tests indicate that we cannot analyze the full sample without adjusting for imbalances (see Appendix A Table A.1). Randomization within each subgroup (Table 2) appears well balanced, suggesting that it was successful. No adverse events or unintended effects were reported or observed in either the intervention or control group during the study period.

Table 3 presents the results as average marginal effects for both subgroups combined.

**Table 3:** Marginal effects of intervention, checkup before, and individual characteristics on checkup estimated by probit regression for the entire sample.

Variable name	Coefficient	Standard Error	CI
Intervention	0.035*	(0.015)	[0.006, 0.064]
Previous checkup	0.521***	(0.018)	[0.486, 0.556]
Male	0.009	(0.015)	[−0.020, 0.038]
Age [Reference *(20–39)*]			
40–59 years	−0.006	(0.069)	[−0.141, 0.129]
60+ years	0.060	(0.068)	[−0.073, 0.193]
Years with T2DM [Reference *(<1 year)*]			
1–2 years	−0.057	(0.032)	[−0.120, 0.006]
3–10 years	−0.158***	(0.029)	[−0.215, −0.101]
11+ years	−0.156***	(0.027)	[−0.209, −0.103]

Note. The standard errors are indicated in parentheses. ^⁎^*p* < 0.05, ^⁎⁎^*p* < 0.01, and ^⁎⁎⁎^*p* < 0.001. 95% CIs for marginal effects are included. CIs are approximate due to rounding of reported proportions. A total of 212 observations were dropped due to missing data on “male” and “Age.”

The effect of the intervention is statistically significant, corresponding to a 3.5 percentage point increase in comprehensive checkups. This represent a 4.8% increase relative to the initial attendance rate of 73%. Whether a person had previously undergone an annual checkup has strong predictive power.

The covariates may not fully remove the effect of the imbalance between the two subgroups. For instance, individuals with access only to SMS may be less likely to respond to nudges than those with access to both email and SMS. In a second analysis, we therefore compare the difference in checkups between the control and intervention groups within each subsample separately. Table 4 presents the results. We reject the hypothesis that checkup rates are equal between the control and intervention groups in Group 1; the difference of 4.2 percentage points is statistically significant. For Group 2, the difference of 3.7 percentage points is not statistically significant.

**Table 4:** Results of the chi-square test for each subgroup.

	Group 1: Email and SMS			Group 2: Only SMS		
	Intervention	Control	Diff (CI)	Intervention	Control	Diff (CI)
Checkup proportion	69.8	65.5	4.2* (0.6–8.0)	69.5	65.8	3.7 (−2.1–9.5)
N	1217	1201		505	488	

Note. * *p* < 0.05. 95% CIs for group differences calculated using a two-proportion z-test. CIs are approximate due to rounding of reported proportions.

## 4 Discussion and conclusion

### 4.1 Discussion

In aggregate, the estimated marginal effect of the intervention on the probability of undergoing a comprehensive checkup is 3.5 percentage points. For Group 1, which received an email followed by an SMS, the estimated effect is 4.2 percentage points, whereas the effect for Group 2 is not statistically significant.

Although an effect of 3.5–4.2 percentage points may appear modest, it represents a meaningful increase in absolute terms when scaled to a broader population. With approximately 250,000 people living with T2DM in Norway, and assuming that roughly 30% attend comprehensive annual checkups, this effect size translates into an additional 2625 individuals completing their annual checkup. Beyond the relatively minor administrative costs, the primary cost of such a campaign consists of SMS messaging fees. Prior research has documented improvements in quality of life attributable to reductions in modifiable cardiovascular risk factors [17]. Furthermore, studies evaluating disease management programs for newly diagnosed T2DM patients have estimated an average gain of 2.55 days in perfect health per year [23]. Extrapolating this figure to the present context, the implied aggregate benefit amounts to nearly 19 life-years in perfect health gained annually across the entire T2DM population. Because the health gain is likely to be smaller among individuals who have lived with T2DM for a long time, this estimate should be interpreted as an upper bound.

Firm projections of effects following the scaling of interventions have been criticized by several authors [[24], [25]]. NDA members differ from the general T2DM population in ways that affect their responsiveness to interventions. On the one hand, because NDA membership signals an interest in one's own health, the sample may be more responsive to information than the wider T2DM population. On the other hand, the high proportion of members with a previous checkup may mean that the potential for a further increase is limited.

Despite limited generalizability of our results to the T2DM population, we would argue that our design mimics real-world settings for patient organizations and offers advantages over population-wide studies in terms of feasibility. Because the NDA secretariat communicated with its members through an established structure, the administrative costs of the study were kept to a minimum. An alternative approach would be to identify the entire population of people with T2DM using their ICPC2 (International Classification of Primary Care, 2nd Edition) diagnoses as recorded by their GPs. This group would then have to be contacted to obtain their consent. In addition to substantial administrative costs, it is unclear whether the proportion of people who provide consent would be representative of the entire population with T2DM.

Regarding our experimental design, we cannot rule out spillover from the intervention group to the control group (i.e., within households or in closed Facebook groups). The control group would then have access to information intended only for the intervention group; in the presence of such spillovers, we would underestimate the effect of the intervention. The stratification of our sample into two groups was based on the availability of contact information, which made the comparison of the two interventions challenging. If the telephone numbers provided were for landlines rather than mobile phones, we would also potentially have underestimated the effect; the same applies to deceased individuals. However, the NDA advised us that the use of landline numbers and the number of deceased members would be limited. In any case, we argue that an undelivered SMS reflects a real-world setting. A further limitation is that we estimated the effect among the people who responded to the questionnaire, who may also be more likely to respond to the interventions. This restriction corresponds to estimating the average treatment effects on the treated rather than the average treatment effect.

### 4.2 Innovation

This study contributes to the literature on innovations in public health by demonstrating how disseminating information about recommended disease management can enable patients to take action to improve their health. Although patient reminders can improve appointment attendance in outpatient care [5], little research has examined the specific benefits of attending these appointments. In addition to the simple nudge, we directed patients to a small online educational component.

In specific terms, the study's innovation lies in introducing and rigorously testing a low-cost, demand-side nudge delivered through an existing national patient organization's established communication infrastructure rather than through providers or health authorities. The intervention combines channel design (email plus SMS reminder versus SMS only) with a brief online educational video hosted on a closed NDA webpage, thereby blending a classic “nudge” with a lightweight “boost” component that informs patients about recommended disease management and the benefits of comprehensive annual checkups with their GPs. Outcomes were tracked using web questionnaires linked to the study arms.

In more general terms, the study extends the growing literature on SMS and email reminders and digital nudges — often focused on missed appointments, vaccinations, medication adherence, or HIV/STD risk reduction — to the context of guideline-recommended annual diabetes checkups within a universal GP scheme. By demonstrating that a simple, scalable campaign with essentially zero marginal cost per additional patient reached can increase comprehensive checkup attendance by 3.5–4.2 percentage points, the study shows how digital nudges can complement provider-side measures such as financial incentives and clinical software.

### 4.3 Conclusion

The societal cost of T2DM in Norway is estimated at NOK 30 billion (USD 3.5 billion) per year [26]. Early detection of risk factors for disease complications is crucial not only for improving the quality of life of people with T2DM but also for reducing these societal costs. For most diagnostic tests included in a comprehensive annual checkup, the health benefits clearly outweigh the costs [27], indicating that measures to increase attendance are sound investments. Innovative nudging mechanisms, such as the intervention evaluated in this study, are inexpensive to implement and further strengthen the case for measures that increase attendance at comprehensive annual checkups.

## Declaration of generative AI and AI-assisted technologies in the manuscript preparation process

During the preparation of this work the authors used Claude and Chat-GPT in order to suggest improvement of language formulations. After using this tool/service, the authors reviewed and edited the content as needed and takes full responsibility for the content of the published article.

## CRediT authorship contribution statement

**Ole Kristian Aars:** Visualization, Validation, Software, Methodology, Formal analysis, Data curation, Conceptualization, Writing – review & editing, Writing – original draft. **Tor Iversen:** Visualization, Validation, Supervision, Software, Project administration, Methodology, Investigation, Formal analysis, Conceptualization, Writing – review & editing, Writing – original draft.

## Ethical considerations

The Norwegian Agency for Shared Services in Education and Research has evaluated the study. The agency deemed it unnecessary to conduct a full ethical review due to the use of anonymized data.

Data will be made available on reasonable request

## Declaration of competing interest

The authors declare the following financial interests/personal relationships which may be considered as potential competing interests:

**Table:** 

Ole Kristian Aars reports financial support was provided by The research council of Norway. If there are other authors, they declare that they have no known competing financial interests or personal relationships that could have appeared to influence the work reported in this paper.

## Figures and Tables

**Table A.1:** Descriptives according to subgroups

	Group 1 (email and SMS)	Group 2 (only SMS)	Difference
Previous checkup	0.714	0.774	−0.060*
Male	0.506	0.466	0.040
Age			*
20–39 years	0.015	0.005	0.010
40–59 years	0.229	0.122	0.107
60+ years	0.756	0.872	−0.116
Years with T2DM			*
< 1 year	0.046	0.005	0.041
1–2 years	0.096	0.031	0.065
3–10 years	0.274	0.157	0.117
11+ years	0.584	0.807	−0.223

Note. We tested each variable: i) proportions using a two-sample test with an asymptotically normally distributed test statistic and ii) categorical variables with a two-sample Kolmogorov–Smirnov test. Equal distributions in Groups 1 and 2 were rejected for most variables at *p < 0.05 using asymptotically normally distributed test statistics and a two-sample Kolmogorov–Smirnov test.

**Table A.2:** Opening rates and questionnaire responses.

Group	Subgroup	Sent (Emails bounced)	Response rate (%)*		Increase in response rate from “before reminder”
			Before reminder	Total after reminder	
1: Email + SMS	Intervention 1.1 (questionnaire 1)	3906 (486)	313 (9%)	1217 (31%)	289%
	Control 1.2 (questionnaire 2)	3907 (515)	302 (9%)	1201 (31%)	298%
2: SMS + SMS	Intervention 2.1 (questionnaire 3)	2335 (N/A)	268 (11%)	505 (22%)	88%
	Control 2.2 (questionnaire 4)	2336 (N/A)	259 (11%)	488 (21%)	88%

Note. * The denominator is “sent” for all subgroups, except for “before reminder” for Group 1, where bounced emails are subtracted.

**Fig. A.1:**
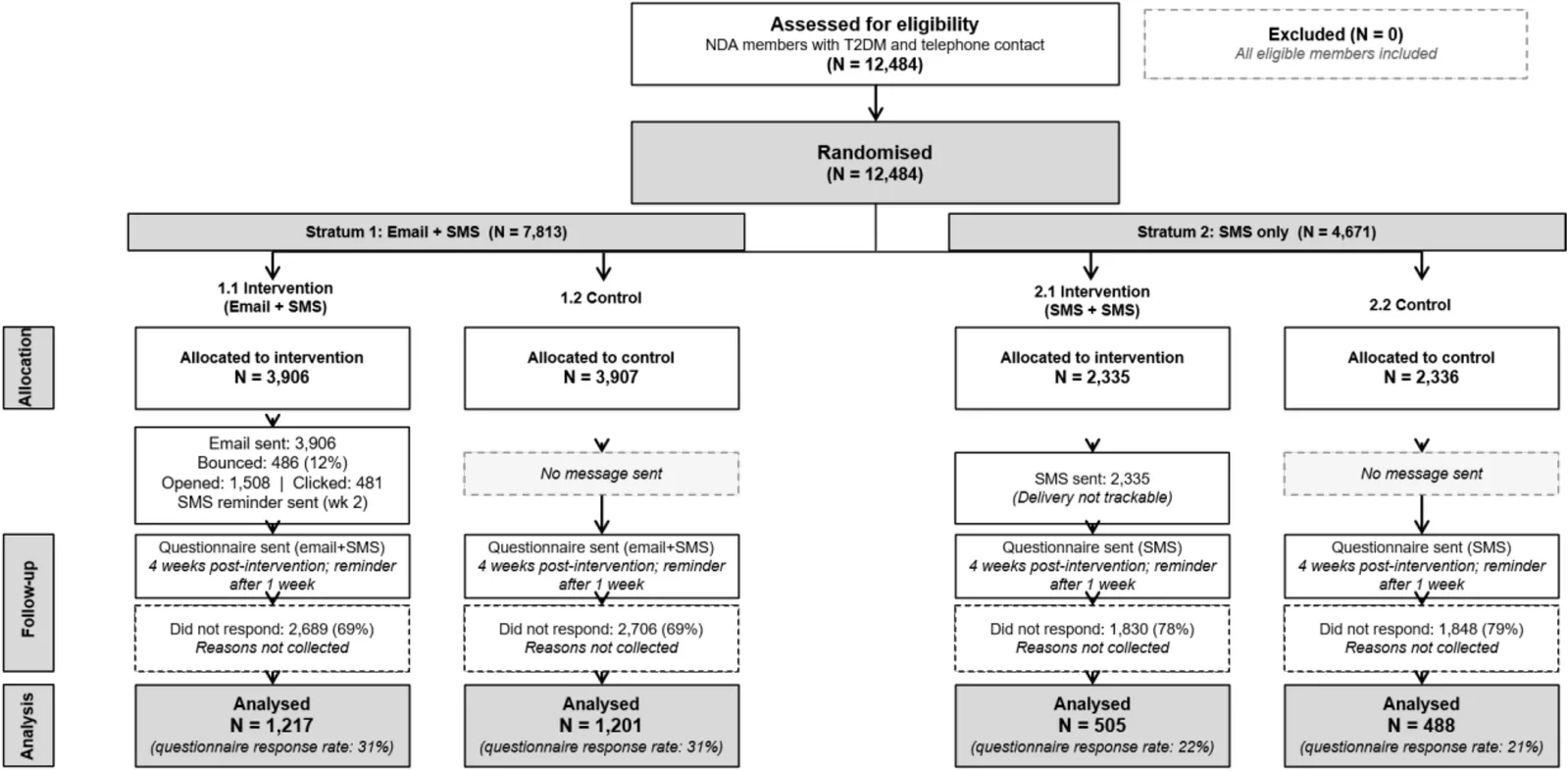
Experimental design. Notes: SMS delivery for Group 2 was not trackable. Control groups received no intervention message.

**Table:** 

Posed if response dummy =	Question	Response dummy
Posed to all	**Have you visited your GP in the last 12 months?**	
	Yes	1
	No	2
	No, but I scheduled an appointment	3
	Don't remember	4
3	**What is the purpose of the appointment?**	
	Annual checkup^a^	5
	Something different	6
1	**Was one of these visits an annual checkup?**	
	Yes^a^	7
	No	8
	Don't remember	9
1	**During the last GP visit, which of the following were checked?**^a^	
	Inspection of feet	10
	Smoking habits	11
	Blood pressure	12
	Urine test	13
	None of the above	14
	Don't remember	15
8 9	**Have you asked your GP about undertaking a checkup?**	
	Yes, I have asked for an appointment^a^	16
	No	17
	Yes, but the doctor said it was unnecessary^a^	18
Posed to all	**Have you been to an annual checkup, prior to the last 12 months?**	
	Yes^b^	19
	No	20
	Don't remember	21
20 21	**During a previous GP visit (prior to the last 12 months), which of the following were checked?**^b^	
	Inspection of feet	22
	Smoking habits	23
	Blood pressure	24
	Urine test	25
	None of the above	26
	Don't remember	27
Posed to all	**I am**^c^	
	Male	28
	Female	29
Posed to all	**My age is**^c^	
	20–39 years	30
	40–59 years	31
	60+ years	32
Posed to all	**How long have you been diagnosed with diabetes?**^c^	
	Less than a year	33
	1–3 years	34
	3–10 years	35
	More than 10 years	36

a Any of these responses were coded as “1” in the “checkup” variable. For specific diagnoses, people had to say yes to all questions in 10–13.

b Any of these responses were coded as “1” in the “previous checkup” variable. For specific diagnoses, people had to say yes to all questions in 22–25.

c Non-mandatory questions.

**Fig. C.1:**
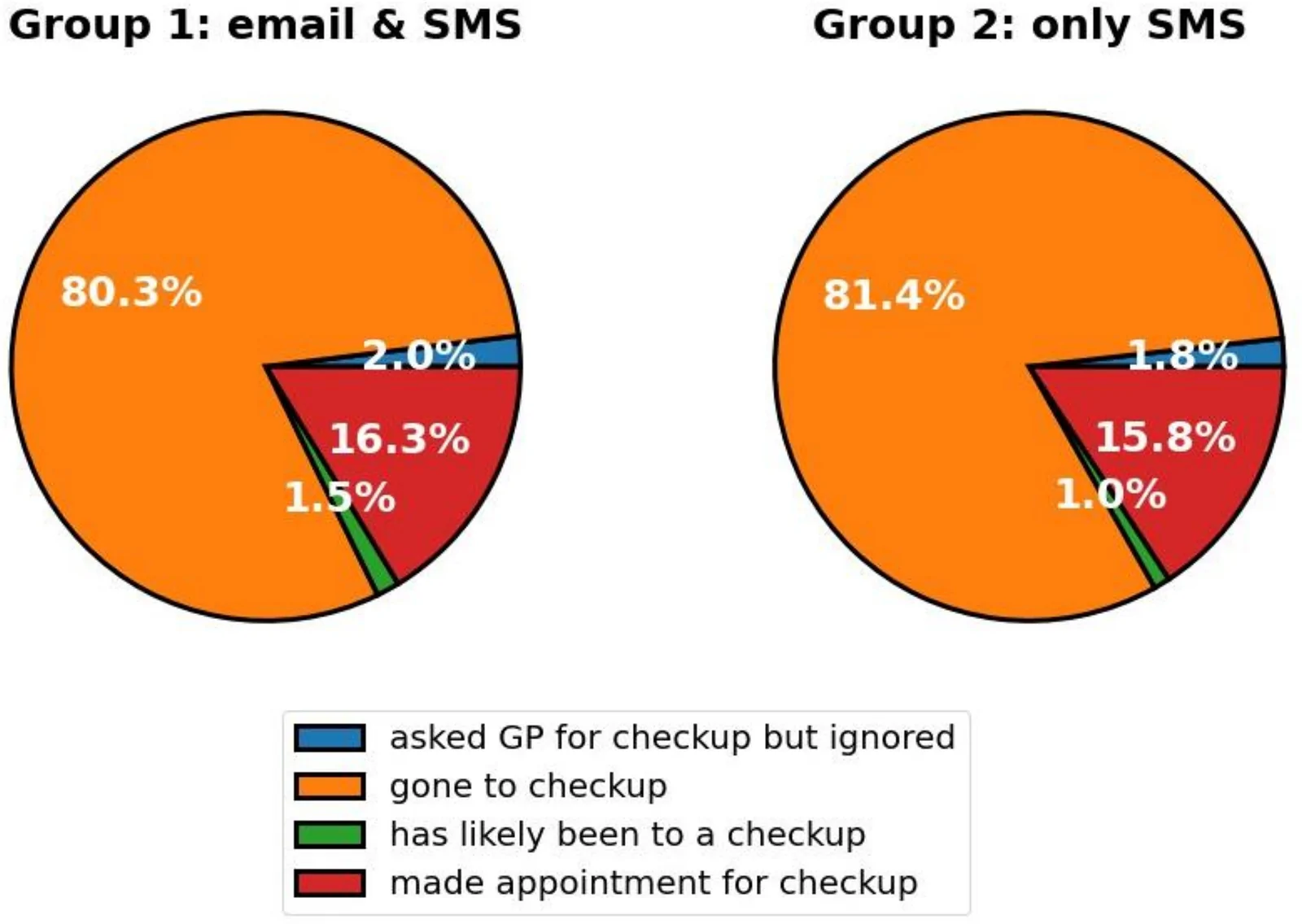
Breakdown of dependent variable “checkup” for each subgroup. * “Has likely been to a checkup” =1 if responded “yes” to all questions 10–13.

**Fig. C2:**
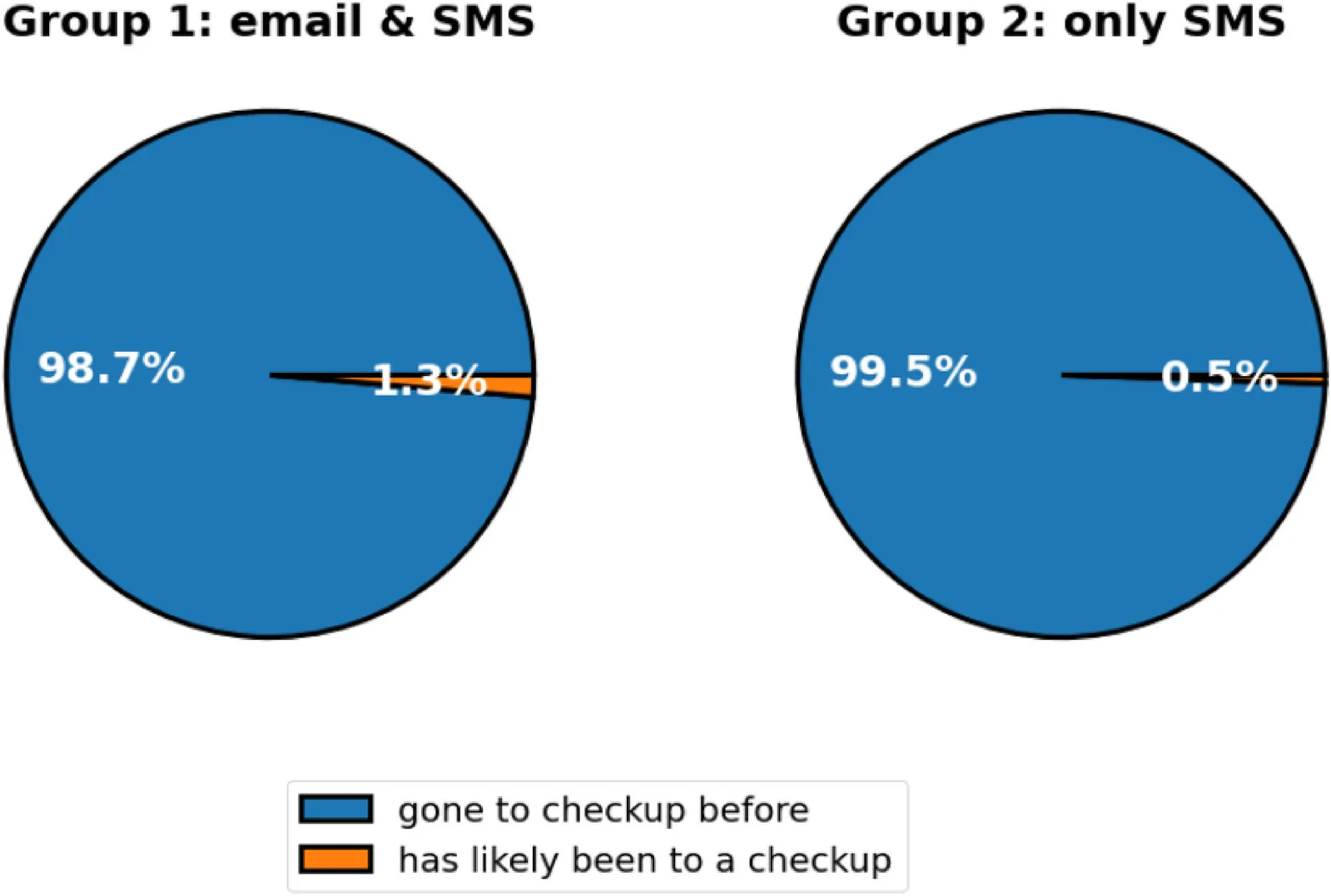
Breakdown of the independent variable “Previous checkup.” * “Has likely been to a checkup” =1 if responded “yes” to all questions 22–25.

## Data Availability

Data were collected via the Norwegian Diabetes Association's (NDA) membership systems and were in the joint custody of the NDA and the research team. Researchers wishing to replicate or extend this work are encouraged to contact the NDA directly (www.diabetes.no).

## Appendix B Questionnaire

Several questions were asked based on the previous responses (refer to the left and right columns for guidance). Beyond questions related to personal information, all other questions had to be answered before the questionnaire was submitted. The asterisks indicate which questions formed the basis of the *checkup* and *previous checkup* variables.

Questions posed.

## Appendix C Breakdown of aggregated variable

In defining our dependent variable, we defined people who were likely to have gone to a checkup or planned one due to the intervention. Naturally, we counted all those who had gone to a checkup and those who had made an appointment. The latter is included to reflect that, given the short follow-up period from intervention to questionnaires, some people may not have had time to visit their GP but are still motivated to undertake a checkup. Therefore, we included only those who had made an appointment for a checkup. We also included those who had unknowingly undergone checkups. We were advised by NDA that many people are not necessarily aware that a given doctor visit involves diabetes “comprehensive checkup.” Accordingly, we categorized visits in which tests included in a standard checkup were performed (urine and blood pressure tests, smoking counseling, and foot examination). Lastly, there is a small proportion of people who have asked their GP for a checkup but have had their requests ignored. We argue that the intervention affected this group and included them accordingly. A breakdown of the variables is provided below.

Similarly, to the dependent variable, we define “previous checkups” as those who have either stated that they have been to an annual checkup, or those we believed to have had one. In the latter case, we included those who underwent tests included in a standard checkup (urine and blood pressure tests, smoking counseling, and examination of feet). We provided a breakdown of the variables in the figure.

## Appendix D Supplementary data

Supplementary material 1 CONSORT 2025 Checklist.

Supplementary material 2 Response from Regional Committees for Medical and Health Research Ethics.

## Footnotes

1 Appendix B displays the list of questions asked.

2 As the Norwegian Diabetes Association could not share member records, they conducted the randomization based on an excel model developed by the authors that randomly assigned members to either group. Within each stratum, simple randomization was applied: members were assigned a random number using the RAND() function, the list was sorted and split at the midpoint into intervention and control groups.
