# Supplementary material for: Can nudging increase the uptake of recommended health services?
Source: PEC Innov. 2026 Sep 26;9:100508. doi: 10.1016/j.pecinn.2026.100508 (PMC13654448; doi:10.1016/j.pecinn.2026.100508)
Supplement: Supplementary file 1 [file mmc2.pdf]

Region:	Saksbehandler:	Telefon:	Vår dato:	Vår referanse:
REK sør-øst A	Anne Schiøtz Kavli	22845512	04.01.2021	218637
			Deres referanse:	

Ole Kristian Aars

218637 Bemyndiggjøring av pasienter

Forskningsansvarlig: Universitetet i Oslo

Søker: Ole Kristian Aars

Søkers beskrivelse av formål:

Mange pasienter med kroniske sykdommer og lidelser går ikke til kontroller som er anbefalt gjennom faglige retningslinjer. Det kan være mange grunner til dette, men en av forklaringene kan være at pasientene ikke selv vet om hva anbefalt praksis er, og at det ikke er noen automatikk i innkalling til kontroll. I denne studien skal vi undersøke effekten av å gi pasienter informasjon om optimal behandling. Spesifikt skal vi gi gjennomføre en informasjonskampanje hvor vi oppfordrer til årskontroll hos fastlegen, og deretter undersøke hvorvidt dette fører til økt bruk av årskontroll. For å undersøke dette skal vi sende ut informasjon om årskontroll til halvparten av relevant medlemsmasse til en pasientorganisasjon vi samarbeider med - vi kaller disse "gruppe 1". De som ikke mottar informasjon (i første omgang) kaller vi "gruppe 2". Om lag 2-3 måneder etter informasjonskampanjen sender vi ut en spørreundersøkelse til alle medlemmene, der vi blant annet spør om de har vært til kontroll. På bakgrunn av dette kan vi sammenligne om andelen er høyere i gruppe 1 enn gruppe 2. Deltakelse i spørreundersøkelsen er frivillig, og vi vil ikke be om personlige data som del av studien.

REKs vurdering

Vi viser til skjema for framleggingsvurdering mottatt 9.12.2020 angående prosjektet «Bemyndiggjøring av pasienter». Framleggingsvurderingen er vurdert av leder REK sør-øst A.

Formålet med prosjektet er å undersøke om bedre informasjon til pasienter med kroniske sykdommer vil føre til at flere pasienter går til kontroll. Om lag 216 000 personer har diabetes type 2 (T2D) i Norge, men kun 30 % av disse går til årskontroll. I dette prosjektet vil man lage en informasjonsvideo med oppfordring til å gå til årskontroll. Halvparten av Diabetseforbundets medlemmer vil få et nyhetsbrev med informasjon om årskontroll samt en sms med påminnelse en etter en uke. Den andre halvparten av medlemmene vil være kontrollgruppe. 2-3 mnd senere vil det sendes et spørreskjema til alle medlemmer. Spørsmålene vil være identiske, men landingssidene for svarinnhenting er tre ulike, en for de i intervensjonsgruppen som åpnet nyhetsbrevet, en for de som ikke åpnet nyhetsbrevet og den siste for kontrollgruppen. På denne måten vil man undersøke effekten av intervensjonen uten å innhente personopplysninger. Spørsmålene i undersøkelsen dreier seg om hvorvidt deltakerne har vært hos fastlege og gjennomført årskontroll siste 12 mnd, før de siste 12 mnd og om de planlegger årskontroller i fremtiden. Det spørres også om

egenaktivitet, alder (grupper på min 10 år), kjønn og bosted (fylke). Opplysningene samles inn via TSDs nettskjema.

Slik prosjektet er beskrevet vil det falle utenfor virkeområdet til helseforskningsloven. Det undersøkes ikke hvorvidt informasjonen fører til forbedret helse, men om om bedre informasjon vil gjøre at flere pasienter gjennomfører årskontroll. Det planlegges å gjennomføre intervensjonen uten å innhente personopplysninger.

Vedtak

Ikke fremleggspliktig

Etter REKs vurdering faller prosjektet, slik det er beskrevet, utenfor virkeområdet til helseforskningsloven. Helseforskningsloven gjelder for medisinsk og helsefaglig forskning, i loven definert som forskning på mennesker, humant biologisk materiale og helseopplysninger, som har som formål å frambringe ny kunnskap om helse og sykdom, jf. helseforskningsloven §§ 2 og 4a. Formålet er avgjørende, ikke om forskningen utføres av helsepersonell eller på pasienter/sårbare grupper eller benytter helseopplysninger.

Prosjekter som faller utenfor helseforskningslovens virkeområde kan gjennomføres uten godkjenning av REK. Det er institusjonens ansvar å sørge for at prosjektet gjennomføres på en forsvarlig måte med hensyn til for eksempel regler for taushetsplikt og personvern.

Vi gjør oppmerksom på at vurderingen og konklusjonen er å anse som veiledende jf. forvaltningsloven § 11. Dersom dere likevel ønsker å søke REK vil søknaden bli behandlet i komitémøte, og det vil bli fattet et enkeltvedtak etter forvaltningsloven.

Med vennlig hilsen

Knut Engedal
Professor dr. med
Leder REK sør-øst A

Anne Schiøtz Kavli
Seniorkonsulent
REK sør-øst

Kopi til: Forskningsansvarlig(e)institusjon(er) og medbruker(e)
