# Supplementary material for: Can nudging increase the uptake of recommended health services?
Source: PEC Innov. 2026 Sep 26;9:100508. doi: 10.1016/j.pecinn.2026.100508 (PMC13654448; doi:10.1016/j.pecinn.2026.100508)
Supplement: Supplementary file 2 [file mmc1.docx]

# CONSORT 2025 Checklist

*Manuscript: Can nudging increase the uptake of recommended health services? The effect of a randomized information initiative targeting diabetes patients*

Based on CONSORT 2025 (Hopewell et al., BMJ 2025;389:e081124)

| **No.** | **Section / Topic** | **CONSORT 2025 Item** | **Location in manuscript** | **How the requirement is met** |
| --- | --- | --- | --- | --- |
| **TITLE AND ABSTRACT** | | | | |
| **1a** | TITLE AND ABSTRACT | Identification as a randomised trial | *Subtitle; Abstract (Methods)* | The subtitle reads 'The effect of a randomized information initiative targeting diabetes patients' and the Abstract Methods state 'We randomized 12,484 NDA members with type 2 diabetes into control and intervention groups.' The study is thereby identified as a randomised trial. |
| **1b** | TITLE AND ABSTRACT | Structured summary of trial design, methods, results, and conclusions | *Abstract* | The abstract is structured with five headings: Objective, Methods, Results, Conclusion, and Innovation. It reports the study design (randomised, two strata), the sample (12,484 NDA members with T2DM), the intervention (informational message with video link), and the key results (3.5 pp overall effect; 4.2 pp for Group 1). |
| **OPEN SCIENCE – Trial registration** | | | | |
| **2** | Trial registration | Name of trial registry, identifying number (with URL) and date of registration | *Methods* | The study was assessed by the Regional Committee for Medical and Health Research Ethics, South-East Norway (REK sør-øst A, reference 218637) on 4 January 2021, prior to commencement. REK determined that the project falls outside the scope of the Health Research Act (ikke fremleggspliktig), as it does not collect personal health data. No formal ethical approval was therefore required. |
| **OPEN SCIENCE – Protocol and statistical analysis plan** | | | | |
| **3** | Protocol and statistical analysis plan | Where the trial protocol and statistical analysis plan can be accessed | *Methods* | No formal written protocol was prepared in advance of data collection. The study design, eligibility criteria, intervention, and planned analyses are fully described in the Methods section of this manuscript. |
| **OPEN SCIENCE – Data sharing** | | | | |
| **4** | Data sharing | Where individual de-identified participant data, statistical code and other materials can be accessed | *Data Availability Statement* | A Data Availability Statement is included. Individual participant data are not available for sharing. Data were collected via the NDA's membership systems and were in the joint custody of the NDA and the research team. Researchers wishing to replicate or extend this work are encouraged to contact the NDA directly (www.diabetes.no). |
| **OPEN SCIENCE – Funding and conflicts of interest** | | | | |
| **5a** | Funding and conflicts of interest | Sources of funding and other support, and role of funders in the design, conduct, analysis, and reporting of the trial | *Acknowledgements* | The Acknowledgements identify the Research Council of Norway (project 288592, 'Modernizing the GP Scheme') as the funder and the NDA as the collaborating organisation. The following statement is included: 'The funders had no role in study design, data collection and analysis, decision to publish, or preparation of the manuscript.' |
| **5b** | Funding and conflicts of interest | Financial and other conflicts of interest of the manuscript authors | *Conflict of interests statement* | A Conflict of Interests statement is included: 'All authors declare no conflicts of interest.' |
| **INTRODUCTION** | | | | |
| **6** | INTRODUCTION | Scientific background and rationale | *Introduction* | The Introduction provides a comprehensive review of the global and Norwegian burden of T2DM, the role of annual checkups in preventing complications, and prior literature on SMS and email reminders and nudging interventions. The rationale for an information-based nudge directed at NDA members is clearly articulated. |
| **7** | INTRODUCTION | Specific objectives related to benefits and harms | *Introduction / Methods* | The hypothesis is explicitly stated: 'We hypothesize that people in the intervention groups increase their attendance because of the messages.' The primary benefit (increased annual checkup rate) is well defined. Given the nature of the intervention — a low-intensity digital information message — no harms were anticipated, as stated in the Methods. |
| **METHODS – Patient and public involvement** | | | | |
| **8** | Patient and public involvement | Details of patient or public involvement in the design, conduct and reporting of the trial | *Methods* | The Norwegian Diabetes Association (NDA), a national patient organisation representing people with diabetes, was a key partner in the design, conduct, and oversight of the trial. The NDA provided the member database, conducted randomisation, sent the intervention messages and questionnaires, and gave substantive input on the outcome definition, including advising on the inclusion of participants who had undergone a checkup without being aware of it. |
| **METHODS – Trial design** | | | | |
| **9** | Trial design | Description of trial design including type of trial, allocation ratio, and framework (superiority, equivalence, non-inferiority, exploratory) | *Methods* | The Methods state: 'Based on the available contact information, we identified two strata and randomized members within each stratum into intervention and control arms, in a parallel-group superiority design.' Table 1 shows near-equal allocation within each stratum (3,906 vs 3,907 in Group 1; 2,335 vs 2,336 in Group 2). |
| **METHODS – Changes to trial protocol** | | | | |
| **10** | Changes to trial protocol | Important changes to the trial after it commenced, including any outcomes or analyses that were not prespecified, with reason | *Methods* | The Methods include the following statement: 'No changes were made to the trial design, eligibility criteria, interventions, or outcomes after the trial commenced.' |
| **METHODS – Trial setting** | | | | |
| **11** | Trial setting | Settings and locations where the trial was conducted | *Methods* | The trial was conducted in Norway in September 2021, in collaboration with the NDA, via the NDA's digital communication infrastructure. Participants were community-dwelling NDA members with T2DM. |
| **METHODS – Eligibility criteria** | | | | |
| **12a** | Eligibility criteria | Eligibility criteria for participants | *Methods* | Eligibility is defined as NDA members who had disclosed a T2DM diagnosis and provided telephone contact details (N = 12,484), of whom 7,813 also provided email addresses. These criteria are clearly described in the Methods and applied consistently across all four subgroups. |
| **12b** | Eligibility criteria | Eligibility criteria for sites and for individuals delivering the interventions (if applicable) | *N/A* | Not applicable. The trial was delivered centrally by the NDA secretariat using existing communication software. No site-level or interventionist-level eligibility criteria apply. |
| **METHODS – Intervention and comparator** | | | | |
| **13** | Intervention and comparator | Intervention and comparator with sufficient details to allow replication; where additional materials can be accessed | *Methods / Appendix A3* | The intervention is described in detail: the content (health benefits of annual checkup, link to a closed NDA webpage with educational video, call to book a GP appointment), the channels (email followed by SMS reminder for Group 1; SMS followed by SMS reminder for Group 2), and the timing (reminder one week after first message). The comparator is no message. The questionnaire used to measure outcomes is reproduced in full in Appendix A3. |
| **METHODS – Outcomes** | | | | |
| **14** | Outcomes | Prespecified primary and secondary outcomes, including measurement variable, analysis metric, method of aggregation, and time point | *Methods / Appendix A3–A4* | The primary outcome 'Checkup' is a binary variable (1/0) defined in the Methods and elaborated in Appendix A4, covering four response situations: having undergone a checkup, having booked an appointment, having received tests included in a standard checkup, or having asked the GP for a checkup. The outcome was assessed via questionnaire four weeks after the first intervention. No secondary outcomes were pre-specified. |
| **METHODS – Harms** | | | | |
| **15** | Harms | How harms were defined and assessed | *Methods / Results* | The Methods state: 'Given the nature of the intervention, no physical, psychological, or other harms were anticipated or systematically assessed.' The Results confirm: 'No adverse events or unintended effects were reported or observed in either the intervention or control group during the study period.' |
| **METHODS – Sample size** | | | | |
| **16a** | Sample size | How sample size was determined, including all assumptions supporting the sample size calculation | *Methods* | A power analysis was conducted using the Wilcoxon signed-rank test. The analysis showed that a minimum detectable effect of 3.3 percentage points would be required to achieve statistical significance at the aggregate level. The decision to send a reminder was made to increase the probability of detecting a significant effect and to mimic real-world settings. The total sample comprised all eligible NDA members with telephone contact details (N = 12,484). |
| **16b** | Sample size | Explanation of any interim analyses and stopping guidelines | *N/A* | Not applicable. The single-wave survey design of this trial does not lend itself to interim analyses or stopping rules. No interim analyses were planned or conducted. |
| **METHODS – Randomisation: Sequence generation** | | | | |
| **17a** | Randomisation: Sequence generation | Method used to generate the random allocation sequence | *Methods / Footnote 2* | Footnote 2 states: 'Within each stratum, simple randomisation was applied: members were assigned a random number using the RAND() function, the list was sorted, and split at the midpoint into intervention and control groups.' The randomisation model was developed by the authors and implemented by the NDA secretariat. |
| **METHODS – Randomisation: Allocation concealment** | | | | |
| **17b** | Randomisation: Allocation concealment | Type of randomisation and details of any restriction (e.g. blocking, stratification) | *Methods / Footnote 2* | Stratified randomisation was used, with two strata defined by available contact information (email and SMS, or SMS only). Within each stratum, simple randomisation was applied with no blocking, using the Excel RAND() function. Allocation concealment was maintained because the NDA secretariat generated the randomisation list and dispatched all messages directly from the membership database, without the research team having access to group assignments prior to dispatch. |
| **METHODS – Randomisation: Implementation** | | | | |
| **18** | Randomisation: Implementation | Who generated the random allocation sequence, who enrolled participants, and who assigned participants to interventions | *Methods / Footnote 2* | The authors developed the Excel randomisation model. The NDA secretariat conducted the randomisation and assigned participants to groups using this model, based on NDA membership records. As stated in Footnote 2: 'the Norwegian Diabetes Association could not share member records, [so] they conducted the randomization based on an excel model developed by the authors.' |
| **METHODS – Blinding** | | | | |
| **19** | Blinding | Who was blinded after assignment to interventions and how; if relevant, description of the similarity of interventions | *Methods* | Due to the nature of the intervention, blinding of participants to their allocation was not possible: the intervention group received messages while the control group did not. Outcome data were self-reported by participants via questionnaire; outcome assessors were therefore also unblinded. This is inherent to this type of pragmatic digital nudge trial. |
| **METHODS – Statistical methods** | | | | |
| **20a** | Statistical methods | Statistical methods used to compare groups for primary and secondary outcomes, including harms | *Methods* | A probit regression model (Equation 1) is specified, with average marginal effects reported for the pooled sample. Chi-square tests for independence are used to compare checkup proportions within each subgroup separately (Table 4). All statistical analyses were performed using Stata version 16. |
| **20b** | Statistical methods | Definition of who is included in each analysis, and how missing data were handled | *Methods / Results* | The analysis is based on questionnaire respondents in each of the four subgroups. This corresponds to estimating the average treatment effect on the treated (ATT) rather than the intention-to-treat (ITT) effect, as acknowledged in the Discussion. A total of 212 observations were excluded from the pooled probit model due to missing data on sex and age, as reported in the note to Table 3. |
| **20c** | Statistical methods | Methods for any additional analyses (e.g. subgroup and sensitivity analyses), distinguishing prespecified from post hoc | *Methods* | Both analyses — the pooled probit regression and the subgroup chi-square tests — were prespecified before data collection, as part of the study design described in the Methods. No post hoc analyses were conducted. |
| **RESULTS – Participant flow** | | | | |
| **22a** | Participant flow | For each group, the numbers of participants who were randomly assigned, received intended intervention, and were analysed for the primary outcome | *Table 1 / Appendix Table A2 / Figure A1* | Table 1 reports randomisation numbers for all four subgroups (total N = 12,484). Appendix Table A2 reports the number of messages sent, bounced emails (486 in Group 1.1; 515 in Group 1.2), and questionnaire respondents for each subgroup (1,217; 1,201; 505; 488). A CONSORT participant flow diagram (Figure A1) documents the flow from randomisation through allocation, questionnaire dispatch, non-response, and analysis for all four subgroups. |
| **22b** | Participant flow | For each group, losses and exclusions after randomisation, together with reasons | *Appendix Table A2 / Table 3 note / Figure A1* | Appendix Table A2 documents bounced emails (486 in the intervention arm and 515 in the control arm of Group 1). Non-response rates are reported for all subgroups (69% in Group 1; 78–79% in Group 2); individual reasons for non-response were not collected, as noted in Figure A1. Additionally, 212 observations were excluded from the pooled probit model due to missing data on sex and age, as reported in the note to Table 3. |
| **RESULTS – Recruitment** | | | | |
| **23a** | Recruitment | Dates defining the periods of recruitment and follow-up for outcomes of benefits and harms | *Methods* | The trial was conducted in September 2021. Questionnaires were sent four weeks after the first intervention, with a reminder after one further week. Exact calendar dates are not available as the original email records were no longer accessible at the time of writing; the relative timeline is fully described in the Methods. |
| **23b** | Recruitment | Why the trial ended or was stopped (if relevant) | *N/A* | Not applicable. The trial followed its planned design and completed normally. No early stopping occurred. |
| **RESULTS – Intervention and comparator delivery** | | | | |
| **24a** | Intervention and comparator delivery | Intervention and comparator as they were actually administered (who delivered, adherence, fidelity) | *Methods / Appendix Table A2 / Figure A2* | Messages were delivered by the NDA secretariat using the NDA's email software and SMS platform. For Group 1, email open rates and click-through rates were tracked using the NDA's email software and are reported in Appendix Figure A1. Bounced emails are documented in Appendix Table A2. SMS delivery tracking was not technically available for Group 2, as noted in the Methods. The control groups received no messages. |
| **24b** | Intervention and comparator delivery | Concomitant care received during the trial for each group | *N/A* | Not applicable. This is a community-based digital information trial. Participants continued routine primary care as normal; there was no differential concomitant care between the intervention and control groups. |
| **RESULTS – Baseline data** | | | | |
| **25** | Baseline data | A table showing baseline demographic and clinical characteristics for each group | *Table 2 / Appendix Table A1* | Table 2 presents baseline characteristics (previous checkup, sex, age group, years with T2DM) for the intervention and control groups within each subgroup, including between-group differences. Balance is confirmed using asymptotically normal test statistics and Kolmogorov-Smirnov tests; the null hypothesis of equal distributions cannot be rejected within subgroups. |
| **RESULTS – Numbers analysed, outcomes, and estimation** | | | | |
| **26** | Numbers analysed, outcomes, and estimation | For each primary and secondary outcome by group: n included in analysis, n with available data, result per group, effect size and 95% CI; for binary outcomes both absolute and relative effect size | *Table 3 / Table 4* | Table 3 reports average marginal effects and 95% confidence intervals from the pooled probit model. The intervention effect is 3.5 percentage points (95% CI: 0.006 to 0.064). Table 4 reports checkup proportions and absolute differences with 95% CIs for each subgroup: Group 1 difference 4.2 pp (95% CI: 0.6 to 8.0 pp); Group 2 difference 3.7 pp (95% CI: −2.1 to 9.5 pp). Both absolute and relative effect sizes are reported (3.5 pp corresponds to a 4.8% relative increase over the baseline attendance rate of 73%). |
| **RESULTS – Harms** | | | | |
| **27** | Harms | All harms or unintended events in each group | *Results* | The Results section states: 'No adverse events or unintended effects were reported or observed in either the intervention or control group during the study period.' |
| **RESULTS – Ancillary analyses** | | | | |
| **28** | Ancillary analyses | Any other analyses performed, including subgroup and sensitivity analyses, distinguishing prespecified from post hoc | *Methods / Results* | Both analyses — the pooled probit regression and the subgroup chi-square tests — were prespecified before data collection as part of the study design. The rationale for conducting both analyses is described in the Methods. No post hoc analyses were conducted. |
| **DISCUSSION** | | | | |
| **29** | DISCUSSION | Interpretation consistent with results, balancing benefits and harms, and considering other relevant evidence | *Discussion / Conclusion / Innovation* | The Discussion interprets findings in line with the results, contextualises the 3.5–4.2 pp effect within the Norwegian T2DM population, considers prior literature on nudging and digital reminders, and addresses both the health benefits and cost implications of the intervention. No harms were observed. The Innovation section situates the contribution within the broader field. |
| **30** | DISCUSSION | Trial limitations, addressing sources of potential bias, imprecision, generalisability, and, if relevant, multiplicity of analyses | *Discussion* | The Discussion addresses the following limitations: possible spillover from the intervention to the control group (leading to underestimation of the effect); selection bias inherent in NDA membership; restriction of the analysis to questionnaire respondents (ATT rather than ITT); potential SMS non-delivery to landline numbers; inability to distinguish pre- and post-intervention GP visits; and concerns about scalability. Generalisability is assessed in both directions, noting that NDA members may be either more or less responsive than the general T2DM population. |
